# *WSL9* Encodes an HNH Endonuclease Domain-Containing Protein that Is Essential for Early Chloroplast Development in Rice

**DOI:** 10.1186/s12284-020-00407-2

**Published:** 2020-07-11

**Authors:** Xingjie Zhu, Changling Mou, Fulin Zhang, Yunshuai Huang, Chunyan Yang, Jingli Ji, Xi Liu, Penghui Cao, Thanhliem Nguyen, Jie Lan, Chunlei Zhou, Shijia Liu, Ling Jiang, Jianmin Wan

**Affiliations:** 1 State Key Laboratory for Crop Genetics and Germplasm Enhancement, Jiangsu Plant Gene Engineering Research Center, Nanjing Agricultural University, Nanjing, 210095 China; 2 Department of Biology and Agricultural Engineering, Quynhon University, Quynhon, Binhdinh 590000 Vietnam; 3 National Key Facility for Crop Gene Resources and Genetic Improvement, Institute of Crop Science, Chinese Academy of Agricultural Sciences, Beijing, 100081 China

**Keywords:** *Oryza sativa*, Chloroplasts, RNA splicing

## Abstract

**Background:**

The plant chloroplast is essential for photosynthesis and other cellular processes, but an understanding of the biological mechanisms of plant chloroplast development are incomplete.

**Results:**

A new temperature-sensitive *white stripe leaf 9***(***wsl9***)** rice mutant is described. The mutant develops white stripes during early leaf development, but becomes green after the three-leaf stage under field conditions. The *wsl9* mutant was albinic when grown at low temperature. Gene mapping of the *WSL9* locus, together with complementation tests indicated that *WSL9* encodes a novel protein with an HNH domain. *WSL9* was expressed in various tissues. Under low temperature, the *wsl9* mutation caused defects in splicing of *rpl2*, but increased the editing efficiency of *rpoB***.** Expression levels of plastid genome-encoded genes, which are transcribed by plastid-coded RNA polymerase (PEP), chloroplast development genes and photosynthesis-related genes were altered in the *wsl9* mutant.

**Conclusion:**

*WSL9* encodes an HNH endonuclease domain-containing protein that is essential for early chloroplast development. Our study provides opportunities for further research on regulatory mechanisms of chloroplast development in rice.

## Background

Rice (*Oryza sativa* L.) is one of the most important food crops in the world and is the main food for more than one-third of the world population. Photosynthesis is a complex process that determines yield. Chloroplasts are semi-autonomous organelles that contain many genes related to photosynthesis (Mandel et al. 1996). Chloroplasts have crucial roles in plant development and growth by utilization of CO_2_ and biosynthesis of carbon skeletons as well as other physiological processes (Sakamoto et al. 2008; Jarvis and López-Juez 2013). Thus, it is essential to identify and clone genes involved in chloroplast development and function.

Nuclear-coded RNA polymerase (NEP) and plastid-coded RNA polymerases (PEP) together determine the biosynthesis and function of chloroplasts (Tiller and Bock 2014). NEP and PEP recognize different types of promoters, but some plastid genes are co-transcribed by NEP and PEP. NEP is a eukaryotic single subunit RNA polymerase encoded by nuclear genes but is located in the plastids (Liere et al*.*^2011^). PEP is a large, complex protein composed of core subunits and additional proteins (Hajdukiewicz et al. 1997). Chloroplast RNAs need to be processed to become functional rRNAs and mRNAs. Many RNA-binding proteins are involved in RNA cleavage, editing, splicing and stability (Tillich and Krause 2010). RNA splicing is a processing event in which the introns of a precursor messenger RNA (pre-mRNA) are removed and its exons are joined. At present, many splicing factors have been isolated and identified, most of which are PPR (pentatriceptide repeat proteins) proteins, chloroplast RNA splicing and ribosome maturation (CRM) domain proteins and some other splicing factors (de Longevialle et al. 2010). In plants the main type of RNA editing is C-to-U, first described in plant mitochondria by Covello and Gray (1989). A similar phenomenon was later observed in plant chloroplasts (Hoch 1991). Many RNA editing factors have been identified, including pentatricopeptide repeat (PPR) proteins, multiple organelle RNA editing factors (MORF), organelle RNA recognition motif (ORRM) containing proteins, protoporphyrinogen IX oxidase1 (PPO1) and organelle zinc finger1 (OZ1). Several PPR genes in rice, such as *OsV4*, *WSL*, *WSL4*, *OsPPR6*, *OsPGL1*, and *WSL5*, function in chloroplast biogenesis, RNA editing, RNA splicing, and chloroplast development (Gong et al. 2014; Tan et al. 2014; Wang et al. 2017; Tang et al. 2017; Xiao et al. 2018; Liu et al. 2018).

HNH motif is about 35 amino acids long and refers to the three most conserved His and Asn amino acid residues in the motif (Galburt and Stoddard 2002; Mehta et al. 2004; Stoddard 2006). HNH proteins include a range of nucleases such as some homing endonucleases, colicins, and restriction endonucleases (Pommer et al. 1999; Ku et al. 2002; Hsia et al. 2004; Saravanan et al. 2004; Shen et al. 2004; Cymerman et al. 2006). HNH motif allows DNA-binding and nuclease activities and plays important roles in many cellular processes, CRN13s contain an endonuclease HNH-like motif and are involved in plant immune responses (Ramirez-Garcés et al. 2015). The chloroplast *psbA* gene of the unicellular green alga *Chlamydomonas reinhardtii* was shown to contain four large group-I introns based on partial sequence analysis (Erickson et al. 1984). *Cr.psbA-4* contains an HNH motif and belongs to the HNH family (Holloway et al. 1999). However, the functions and regulatory mechanisms of proteins containing HNH motifs in rice remain to be elucidated.

In this study, we isolated and characterized rice mutant *white stripe leaf 9***(***wsl9***)**, which showed white-striped leaves at the early seedling stage. The *wsl9* mutant was albinic when grown at low temperature. We isolated the *WSL9* gene by map-based cloning and demonstrated that it encodes an uncharacterized protein containing an HNH domain. Further investigation showed that RNA editing sites in *rpoB* were affected by the mutation and plastid-encoded gene *rpl2* was not completely spliced in the *wsl9* mutant under low temperature.

## Results

### Phenotypic Characteristics of the *wsl9* Mutant

**Fig. 1:**
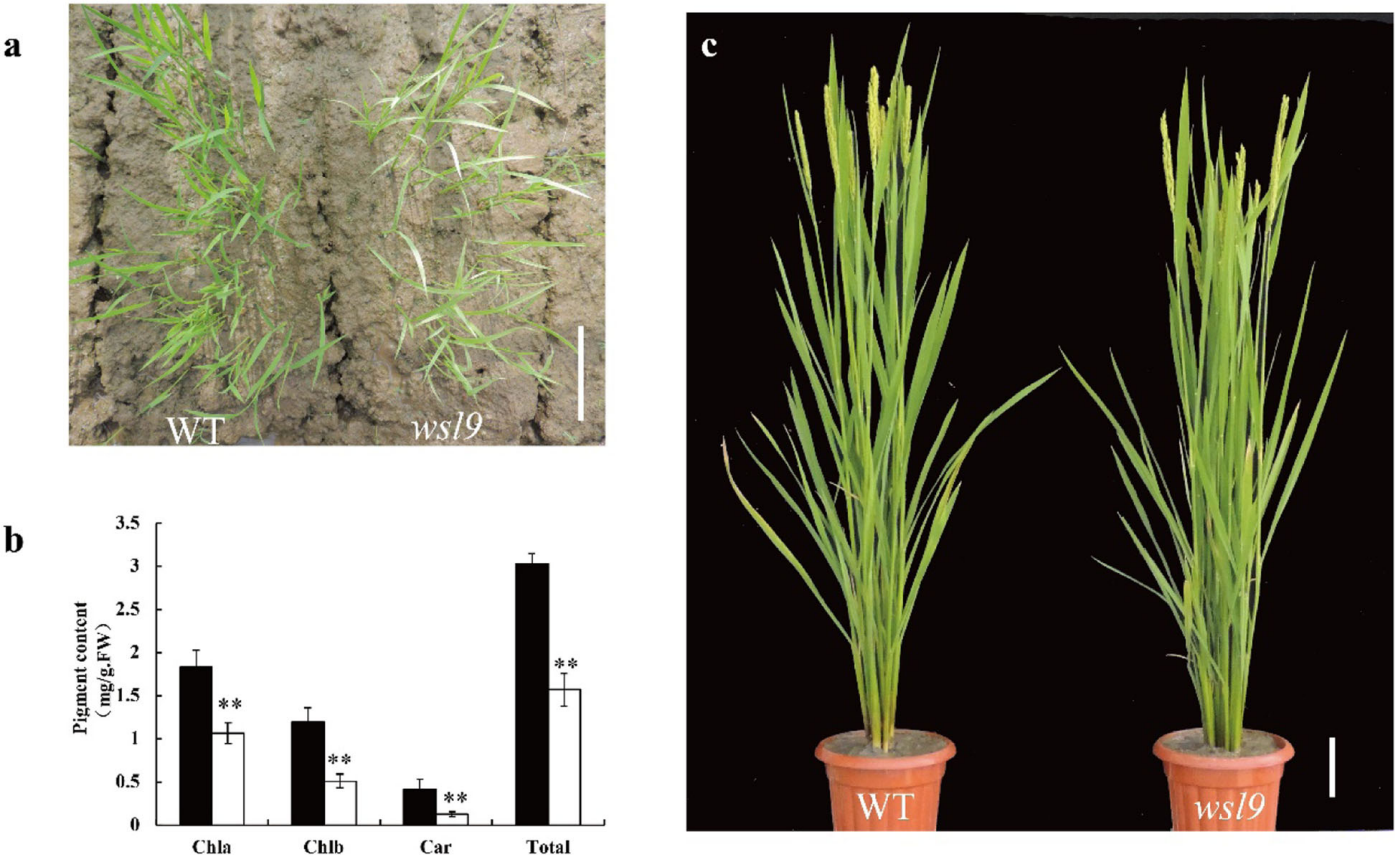
Phenotypic characteristics of wild-type and *wsl9* mutant. **a** Phenotypes of WT (left) and *wsl9* mutant (right) seedlings in the field 2 weeks after seeding. **b** Leaf pigment contents of field-grown WT and *wsl9* seedlings at 2 weeks post-seeding. **c** Phenotypes of WT (left) and *wsl9* (right) plants at 10 days post-heading. (Student’s *t*-tests, **, *P* < 0.01). Scale bars, 10 cm in **a** and **c**

The mechanisms of chloroplast development were studied in a white-striped leaf mutant *wsl9*, identified following ethyl methane sulfonate (EMS) mutagenesis of *japonica* cultivar Ninggeng 3. The leaves of the *wsl9* mutant exhibited white-striped leaves up to the third-leaf stage when planted in the field (Fig. 1a). Chlorophyll (Chla, Chlb) and carotenoid contents were reduced in *wsl9* mutant seedlings (Fig. 1b). Mutant plants become green from the fourth leaf stage and can not be distinguished from the wild type (WT) (Fig. 1c).

**Fig. 2:**
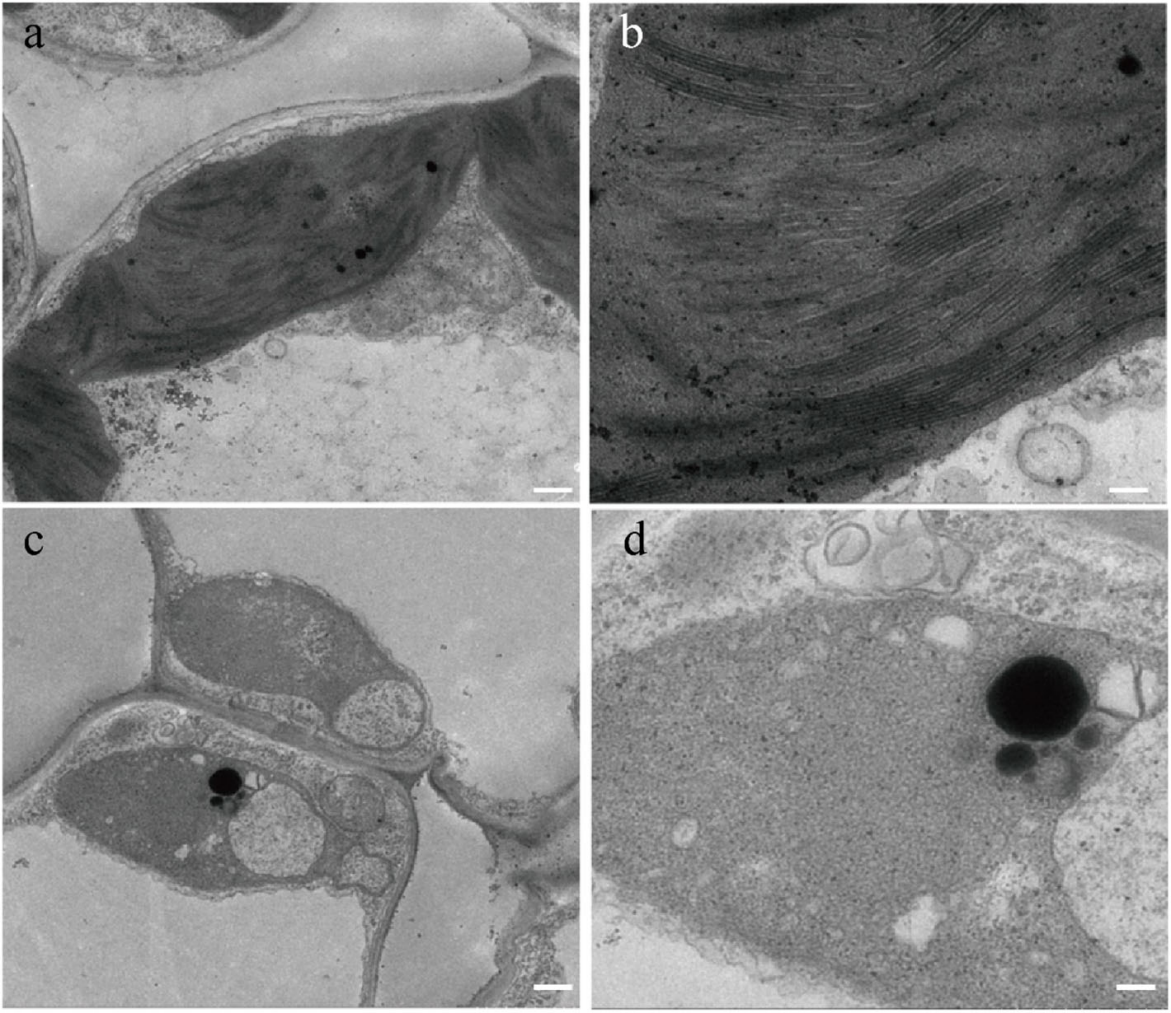
Transmission electron microscope images of chloroplasts in WT (**a**, **b**) and wsl9 mutant (**c**, **d**) seedlings. Scale bar, 0.25 um in a, c; 0.15 um in (**b**, **d**)

We compared the ultrastructures of chloroplasts between white sectors of *wsl9* mutant leaves and WT leaves at the three-leaf stage by transmission electron microscopy (TEM). WT plants had dense and normal grana stacks (Fig. 2a, b) whereas those of the *wsl9* mutant had no organized lamellar structures (Fig. 2c, d).

**Fig. 3:**
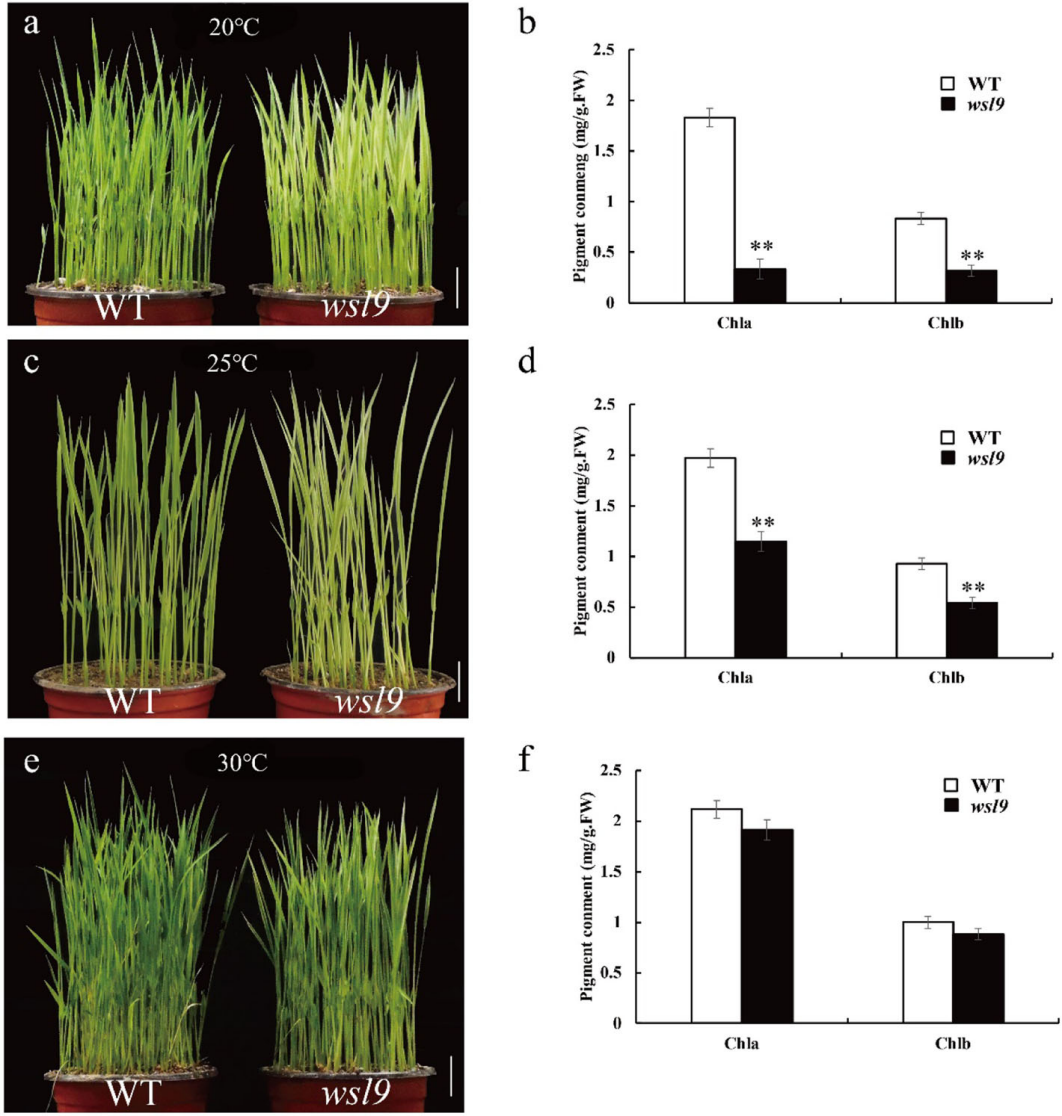
Responses of the *wsl9* mutant to temperature. **a**, **c**, **e** WT and *wsl9* mutant seedlings grown at 20 °C, 25 °C, and 30 °C. Scale bar, 2 cm. **b**, **d**, **f**. Chl a and Chl b contents in L3–3 leaves from WT and *wsl9* mutant seedlings (***n*** = 3) grown in a growth chamber with the same conditions as (**a**, **c**, **e**) (Student’s *t*-tests, ∗∗, *P* < 0.01)

The *wsl9* mutant was sensitive to temperature. We planted WT and the *wsl9* mutant at 20 °C, 25 °C, and 30 °C. When planted at 20 °C, *wsl9* plants were albinic (Fig. 3a), and chlorophyll (Chl) contents were very low (Fig. 3b). At 25 °C *wsl9* plants exhibited white striping and reduced chlorophyll contents (Fig. 3c, d) and at 30 °C the mutant could not be distinguished from the WT (Fig. 3e, f).

### Map-Based Cloning of the *WSL9* Locus

**Fig. 4:**
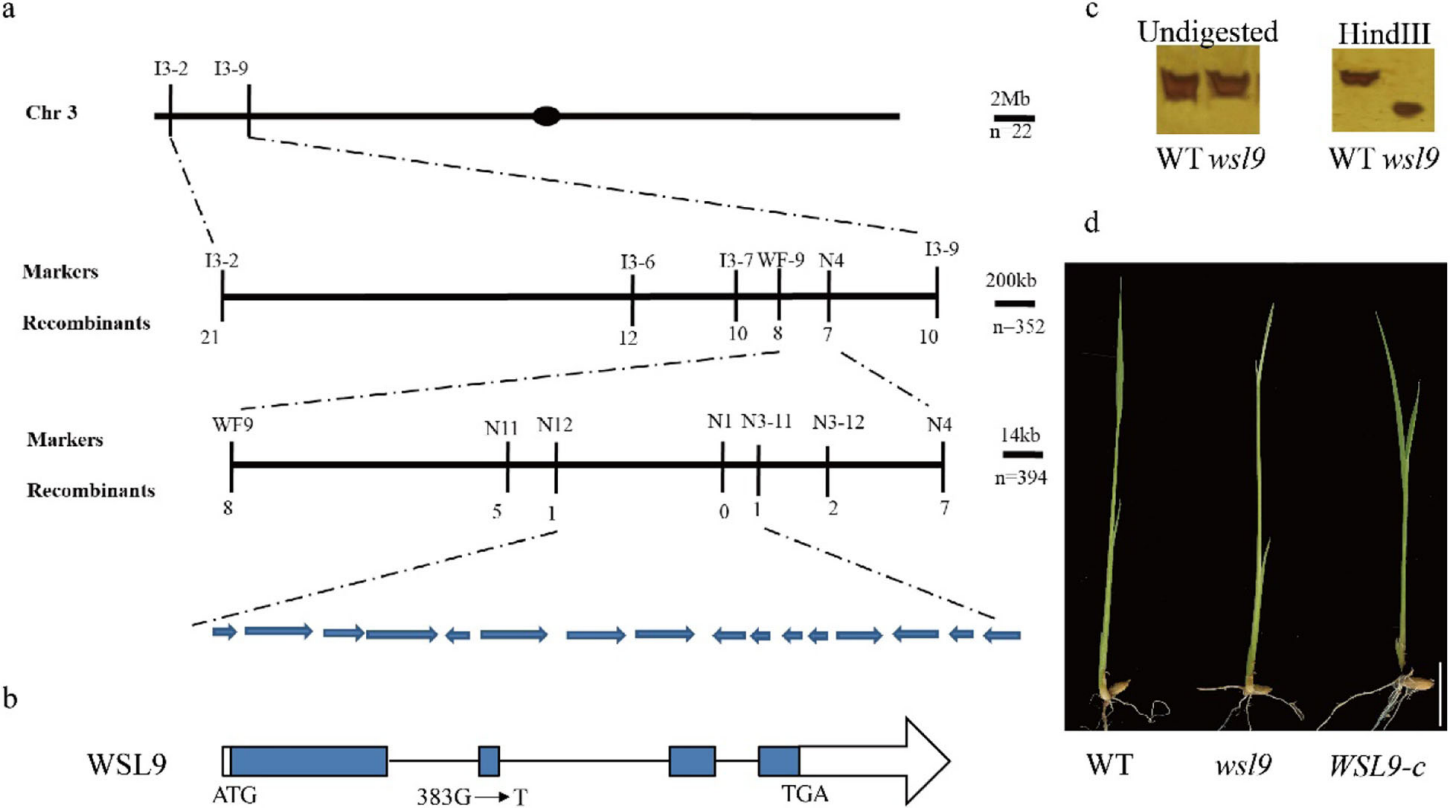
Map-based cloning of the *WSL9* locus and identification of the mutation site in *wsl9* mutant*.***a***WSL9* was mapped to a 89 kb region flanked by N12 and N3–11 in chromosome 3; 16 open reading frames (ORFs) were predicted in the mapped region. **b** Structural model of the *WSL9* gene. ATG and TGA represent the start and stop codons, respectively. Blue boxes indicate exons, lines between boxes indicate introns, and white boxes indicate the 5′- and 3′-UTR. One of the single nucleotide changes resulted in a missense mutation. **c** Verification of the difference between wild-type and *wsl9* genomic DNAs using dCAPs markers. **d** Complementation of *wsl9* by transformation. Scale bar, 2 cm

Genetic analyses indicated that the mutant phenotype was due to a single recessive allele. We isolated the *WSL9* allele by map-based cloning. The *WSL9* locus was initially located to a 89 kb region between InDels N12 and N3–11 on chromosome 3 in an F_2_ population from cross *wsl9* × 93–11. Sixteen open reading frames (ORFs) were predicted in the region (Fig. 4a). We sequenced the entire region in normal and mutant plants and found a SNP (G to T) in ORF *Os03g0169800* at position 383 bp from the ATG start codon. This SNP caused a cysteine to phenylalanine amino acid substitution in the mutant. We used dCAPs markers to confirm the mutant site (Fig. 4c).

To confirm that the mutation was responsible for the *wsl9* mutant phenotype we performed a complementation analysis by transforming the *wsl9* mutant with the *WSL9* allele. Complementation vector p*WSL9*pro:*WSL9*cDNA containing a 2.1 kb upstream sequence and the entire coding region of *WSL9* was constructed and introduced into *wsl9* homozygotes. Nine positive transgenic plants displayed the wild-type phenotype (Fig. 4d). These results confirmed that *Os03g0169800* was the *WSL9* gene.

Analysis of the WSL9 protein in the NCBI database (https://www.ncbi.nlm.nih.gov/) showed that it encodes an uncharacterized protein containing an HNH domain (Additional file 4: Fig. S1a). *WSL9* encodes a protein that contains a HNH motif in its C terminal, so it is possible that WSL9 may have endonuclease activity. Previous reports consider that most characterized HNH proteins possess endonuclease activity. However we failed to detect the endonuclease activity using WSL9 expressed in *E. coli*. Future work will aim to identify if WSL9 require other cofactor to act with its endonuclease activity. BLAST searches found that WSL9 had close homologs in *Arabidopsis thaliana*, *Zea mays*, *Sorghum bicolor*, *Brachypodium distachyon*, and *Gossypium hirsutum* (Additional file 4: Fig. S1b). The functions of all homologs were unclear. As shown in Additional file 4: Fig. S1c the mutation site was conserved in all species.

### Expression Pattern of *WSL9* Gene

**Fig. 5:**
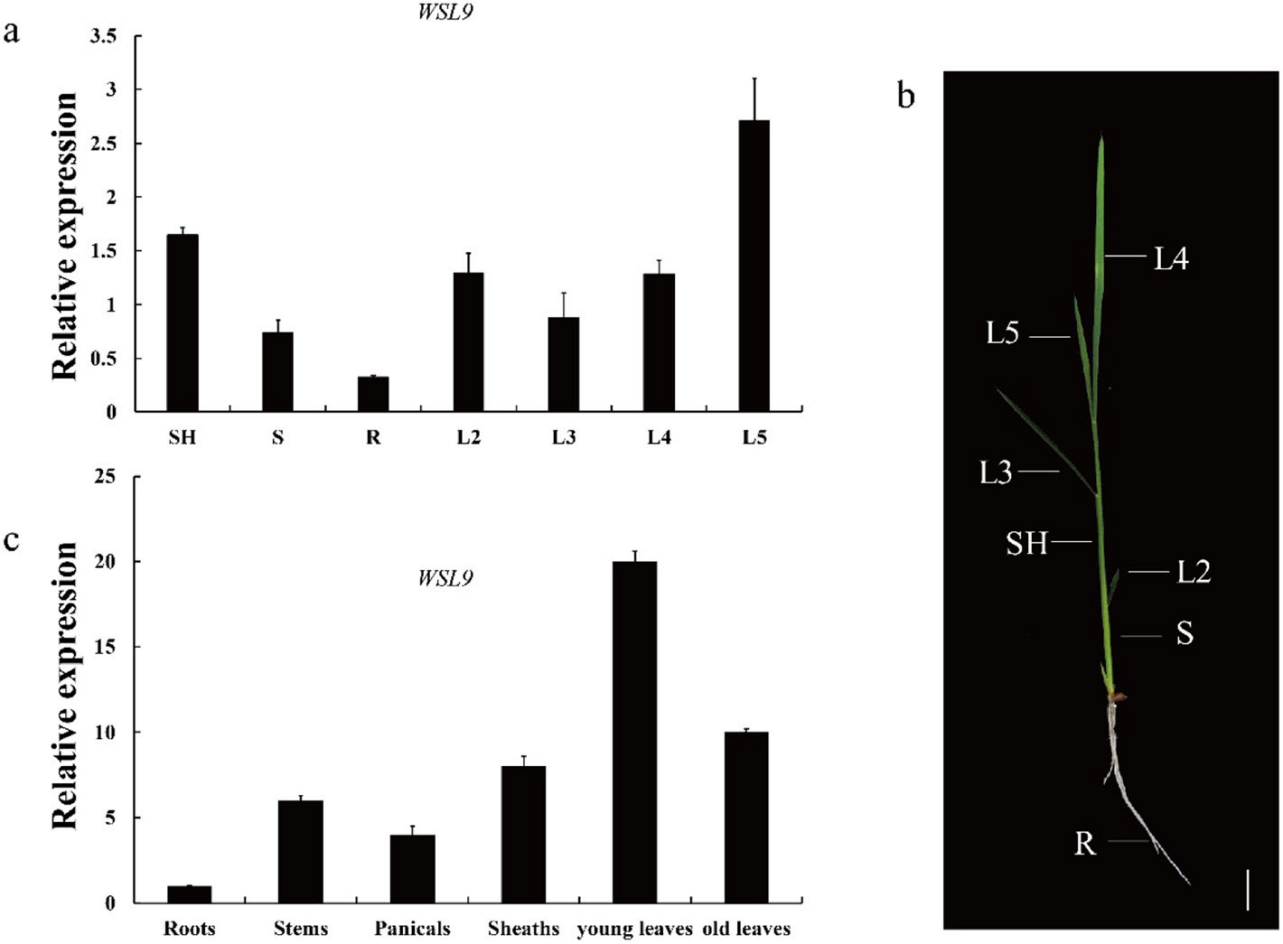
Expression pattern analysis of *WSL9.***a** qRT-PCR analysis of *WSL9* transcripts in the SH, S, R, L2, L3, L4, L5 of wild-type seedlings. Wild-type plants at the five-leaf stage were used for expression analysis. L2–L5 indicate the second to fifth leaves. R, root; S, stem; SH, a 4 cm piece from the bottom of the shoot. **b** Rice seedling with fully expanded fifth leaf. Scale bar, 2 cm. **c** qRT-PCR analysis of *WSL9* transcripts after heading

We analyzed the *WSL9* expression in different sections of leaves at various leaf development stages and the results showed that *WSL9* was most highly expressed in leaf section L5 (Fig. 5a). Using the Rice XPro transcript profling database (http://ricexpro.dna.affrc.go.jp) we found that *WSL9* was expressed in all tissues (Additional file 5: Fig. S2). To certify the data we performed quantitative reverse transcription-PCR (qRT-PCR) using RNA samples from different tissues of WT plants. *WSL9* was expressed in various organs including the young leaves, roots, stems, sheaths, panicles and old leaves. However, *WSL9* transcript was preferentially expressed in young leaves (Fig. 5c).

### The *wsl9* Mutant Is Defective in Plastid Transcription and Synthesis of Chloroplast Proteins

**Fig. 6:**
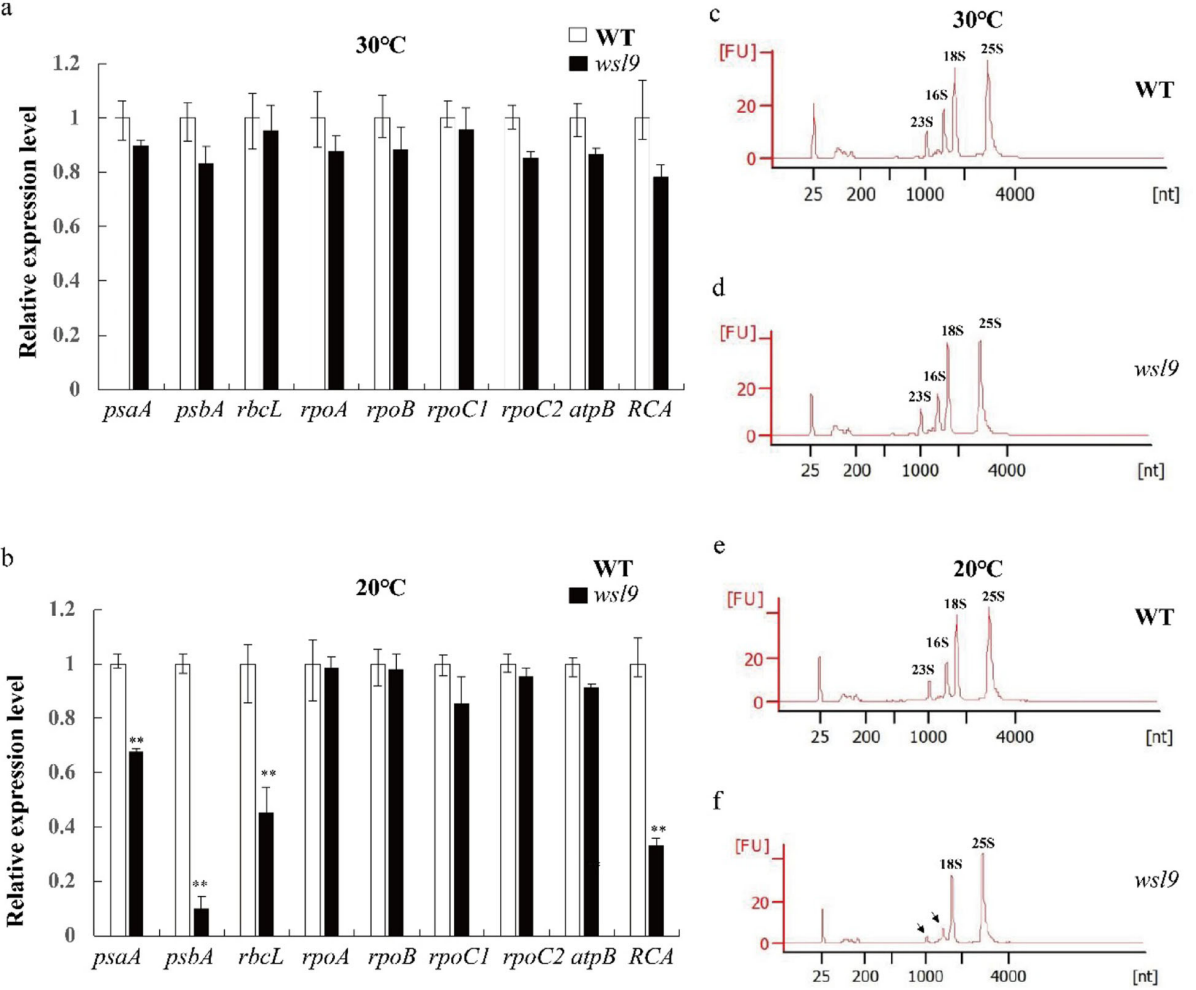
Accumulation of transcripts of chloroplast-associated genes in WT and *wsl9* seedlings. **a**, **b** qRT-PCR analysis of relative expression levels of plastidic encoding genes in wild type and *wsl9* mutant at the third-leaf stage at (**a**) 30 °C and (**b**) 20 °C. Error bars represent SD from three independent experiments. **c-f** rRNA analysis using an Agilent 2100 analyser. RNA was isolated from third-leaf stage WT and *wsl9* mutant seedlings grown at 30 °C and 20 °C. (Student’s *t*-test, ∗∗, *P* < 0.01)

Based on promoter structure, plastid-encoded genes can be defined into three classes (class I, II and III). As previously reported Class I genes are predominantly transcribed by PEP including *psaA*, *psbA* and *rbcL*. *atpB* was selected as a class II gene which is transcribed by both NEP and PEP, and *rpoA, rpoB, rpoC1* and *rpoC2* were selected as class III genes which are exclusively transcribed by NEP (Swiatecka-Hagenbruch et al. 2007). Expression patterns of transcripts of all genes in the *wsl9* mutant and WT were very similar when the plants were grown at 30 °C (Fig. 6a). However, at 20 °C expression levels of class I genes were greatly reduced. In particular, expression of *rbcL*, which encodes the large subunit of Rubisco, was reduced in *wsl9* mutant at 20 °C (Fig. 6b). Rubisco activase (RCA) is a nuclear-encoded, soluble chloroplast enzyme (Andrews 1996; Spreitzer and Salvucci 2002) that regulates the activity of rubisco was also reduced in *wsl9* mutant at 20 °C (Fig. 6b). These results suggested that *wsl9* mutant was defective in PEP activity.

The chloroplast ribosome consists of 50S and 30S subunits. Both subunits are comprised of rRNAs (23S, 16S, 5S, and 4.5S) and ribosomal proteins. We analyzed the composition and contents of rRNAs using an Agilent 2100 instrument in plants grown at 20 °C and 30 °C. 23S and 16S rRNAs were significantly decreased in *wsl9* seedlings grown at 20 °C, but no difference from WT was detected at 30 °C (Fig. 6c-f). Thus the *wsl9* mutant was defective in plastidic ribosome biogenesis under low temperature conditions.

### The *wsl9* Mutation Affects RNA editing and Splicing of *rpl2* Introns 

**Fig. 7:**
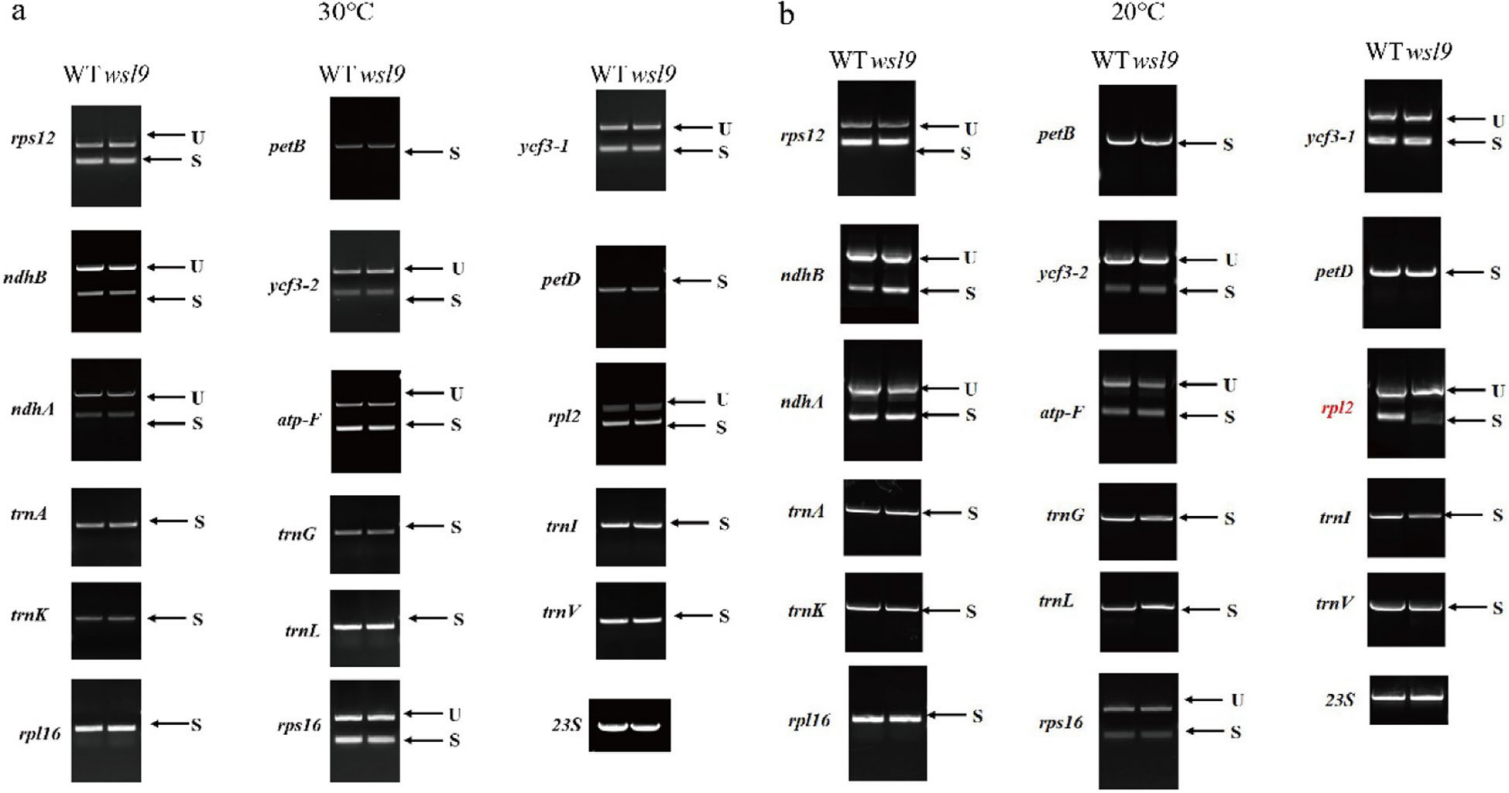
Splicing of chloroplast transcripts in WT and *wsl9* mutant at different temperature. **a** Splicing of chloroplast transcripts in WT and *wsl9* mutant at 30 °C. **b** Splicing of chloroplast transcripts in WT and *wsl9* mutant at 20 °C. Gene transcripts are labeled at the left. Spliced (S) and unspliced (U) transcripts are indicated at the right. RT-PCR was performed with RNA was extracted from WT and *wsl9* mutant at the three-leaf stage. 23S rRNA was used as the reference

**Fig. 8:**
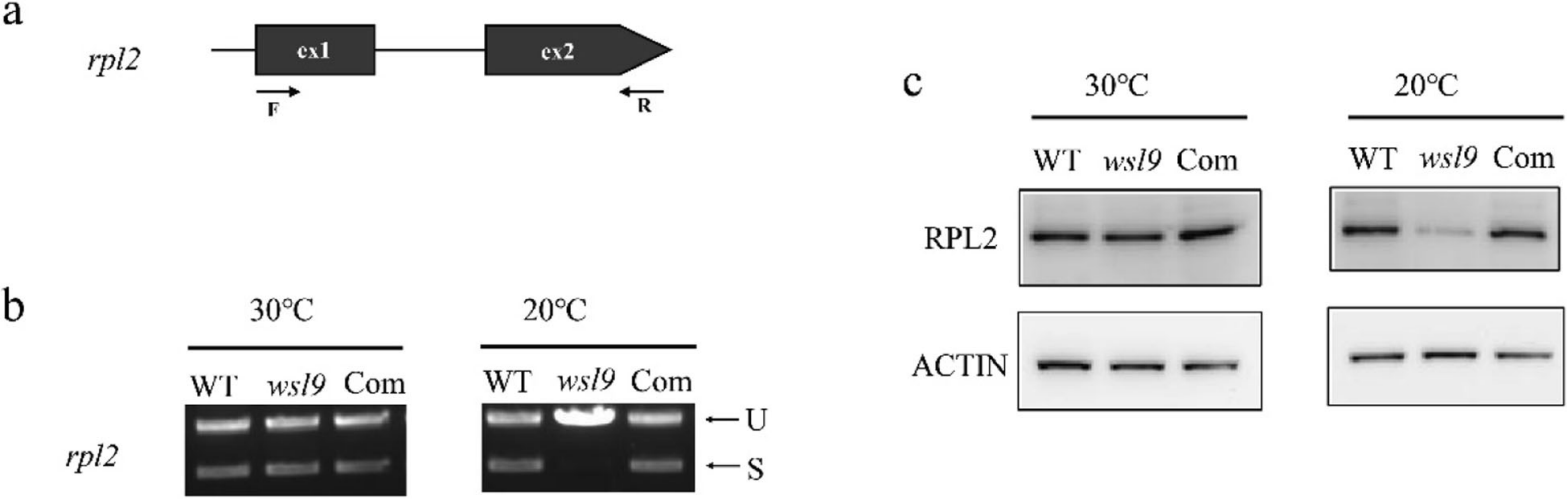
Splicing analyses of *rpl2* in WT, *wsl9* and complemented transgenic plants. **a** Structure of the *rpl2* gene. **b** RT-PCR analyses of *rpl2* transcripts in WT, *wsl9* and complemented transgenic plant under different temperature. Spliced (S) and unspliced (U) transcripts are indicated at the right. **c** Total leaf proteins were analyzed by probing immunoblots with RPL2-specific antiserum. The same amount was immunoblotted with ACTIN antibodies (bottom)

We attempted to determine the function of *WSL9*. Based on a coexpression database (http://ricefrend.dna.affrc.go.jp/) and CREP (http://crep.ncpgr.cn) Module Gene Correlator analysis. We found *WSP1* is one of co-expression genes of *WSL9*. WSP1 showed a high sequence similarity with MORF proteins (Zhang et al. 2017). Although MORFs have only been implicated in RNA editing (Ichinose and Sugita 2016), several examples of intron splicing dependent on RNA editing events have been reported (Hubschmann et al. 1996; Castandet et al. 2010; Farre et al. 2012). In addition, proteins encoded by plastid development-related genes, including ribosomal protein and PPR proteins, were among the co-expressed genes. PPR are also involved in RNA splicing and editing (de Longevialle et al. 2010). On the basis of the results above we investigated whether *WSL9* affected editing at 21 previously identified RNA editing sites in chloroplast RNA (Corneille et al*.*^2000^). The editing efficiency of *rpoB* at C467 and C560 showed a significant increase in *wsl9* mutant compared with WT at 20 °C (Additional file 6 Figure S3), whereas the other 10 genes and corresponding 19 editing sites were unaffected. As expected, the editing efficiency of *rpoB* at C467 and C560 showed marked reductions in complemented plants at 20 °C (Additional file 6 Figure S3**)**. The rice chloroplast genome contains 17 group II introns and one group I intron (Hiratsuka et al. 1989). We carried out RT-PCR using primers flanking the introns, and then compared the lengths of the amplified products between WT and *wsl9* mutant. The chloroplast *rpl2* transcript was spliced with greatly reduced efficiency in *wsl9* mutant compared to WT at 20 °C but not at 30 °C (Fig. 7). The splicing defect was rescued in complemented plant (Fig. 8b). Western blotting showed that RPL2 was present at lower levels in the *wsl9* mutant compared with WT and complemented plant under 20 °C (Fig. 8c). These results suggested that the *wsl9* mutant caused defects in the splicing of *rpl2* especially under low temperature.

### Differentially Expressed Genes in *wsl9* Mutant and Wild Type at Different Temperatures

**Fig. 9:**
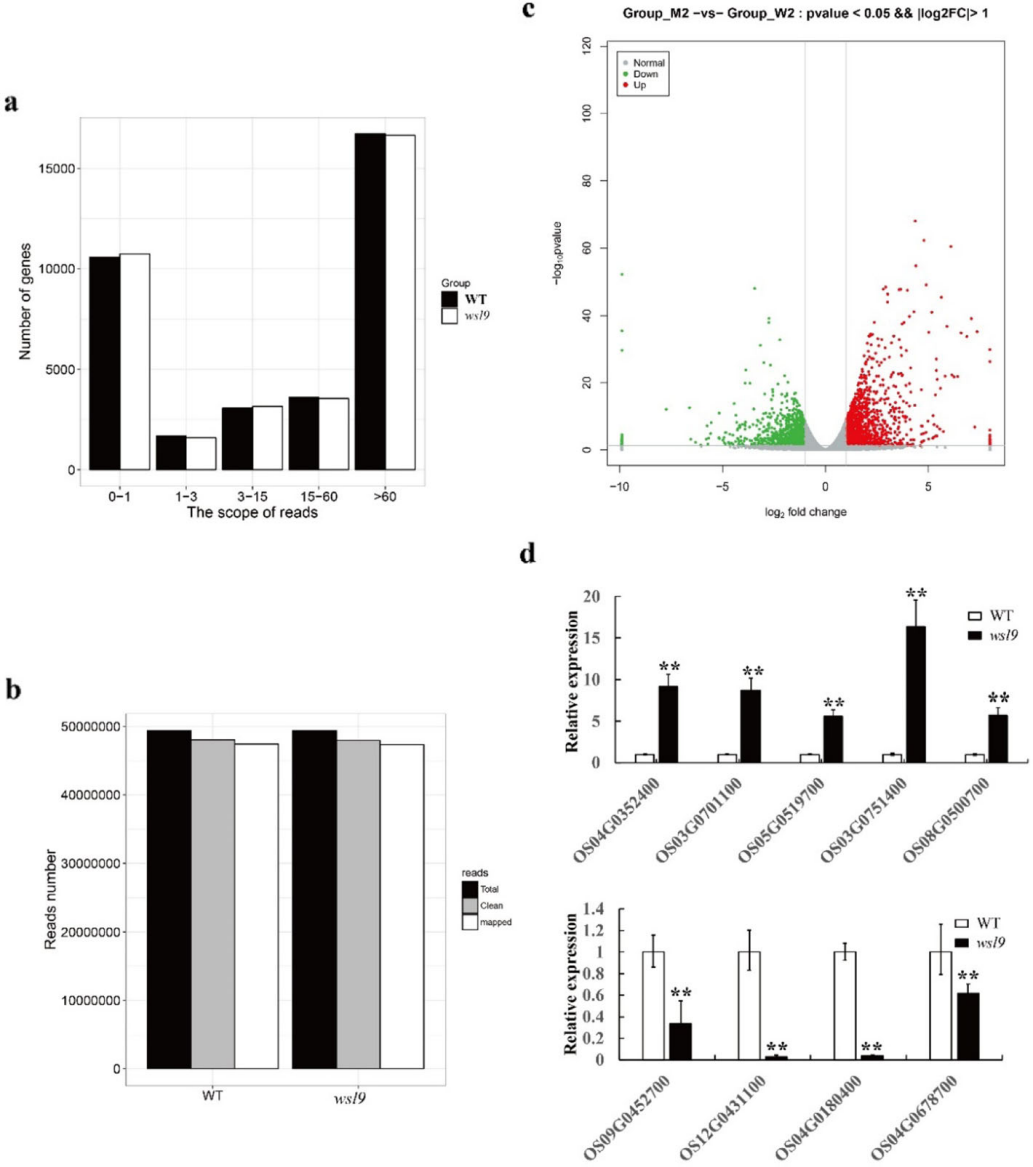
RNA-seq analysis of wild-type and *wsl9* seedlings under 20 °C conditions. mRNA enriched from total RNA isolated from 10-d-old seedlings of WT and *wsl9* mutant using oligo-(dT) was fragmented and reverse-transcribed using random hexamer primers. The library was then constructed and sequenced using an Illumina HiSEquation 2000. **a** Frequencies of detected genes sorted according to expression levels. **b** Read numbers of WT and *wsl9* mutant sequences. **c** Volcano plot showing overall alterations in gene expression in WT and *wsl9* mutant*.***d** qRT-PCR of genes differentially expressed in RNA-seq. Five up-regulated and four down-regulated genes were assayed. Error bars represent SD from three independent experiments. (Student’s *t*-test, **, *P* < 0.01)

We used RNA-seq to explore the effect of *wsl9* on gene expression at different temperatures. A total of 48 million clean reads were obtained from wild type and *wsl9* mutant plants grown at 20 °C condition (Fig. 9a, b). We randomly selected 4 down-regulated and 5 up-regulated genes from 20 °C data to confirm the results of RNA-seq. The data for 30 °C are shown in Additional file 8: Figure. S5**.** There were 1394 up-regulated and 1107 down-regulated genes in *wsl9* at 20 °C, compared with 888 up-regulated and 247 down-regulated genes at 30 °C (Additional file 9: Figure. S6). Go enrichment analysis indicated that genes involved in biological adhesion, rhythmic process, extracellular matrix, structural molecular photosynthesis, PSII, and chloroplast thylakoid were significantly reduced in the *wsl9* mutant at 20 °C (Additional file 9: Figure. S6). These results indicated that the *WSL9* mutation led to change in many physiological processes under low temperature. For example, various chlorophyll synthesis genes, including *HEMA*, *HEML, HEMB, URO-D,CHLH, CHLI, CHLM, CRD, DVR, POR,* and *CHLG* were significantly reduced at 20 °C (Additional file 7: Figure. S4).

## Discussion

Numerous rice leaf color mutants have been reported in rice and numerous other species. Seedling leaf color mutants can be divided into albinic, striped, pale-green, and zebra according to phenotypic pattern. In this study, we identified and characterized a white-striped rice seedling mutant caused by a recessive allele designated as *wsl9*. Under field conditions the striped phenotype was expressed only until the third leaf stage after which the plants developed a normal green phenotype that was identical to the WT. The mutant phenotype was temperature-sensitive under controlled conditions (Fig. 3); it was albinic when plants were grown at 20 °C, exhibited white stripes at 25 °C, and was identical to WT at 30 °C (Fig. 3). The transcription levels of genes associated with Chl biosynthesis and photosynthesis in *wsl9* mutant were affected at the lower temperature. Many previously reported leaf color mutants were also low temperature sensitive, including *v1*, *v2*, *v3*, *str*, *ysa*, *wlp1*, and *val1* (Kusumi et al. 2011; Sugimoto et al. 2007; Yoo et al. 2009; Su et al. 2012; Song et al. 2014; Zhang et al. 2018). *V1* encodes a chloroplast-located NUS1 protein that is essential for establishing the plastid genetic system during early development (Kusumi et al. 2011). *V2* encodes a guanylate kinase that is located in both plastids and mitochondria and functions in chloroplast differentiation (Sugimoto et al. 2007). *V3* and *STR1* encoding the large and small subunits of ribonucleotide reductase, respectively, are mainly involved in DNA synthesis and repair during early leaf development (Yoo et al. 2009). *YSA* encodes a PPR protein with 16 tandem PPR motifs; the *ysa* mutant is albinic before the three-leaf stage but gradually becomes green from the four-leaf stage (Su et al. 2012). *WLP1* encodes a ribosome L13 protein; early seedling leaves and immature panicles of *wlp1* mutant are albinic, and the phenotype is more strongly expressed at low temperatures (Song et al. 2014). *VAL1* is a crucial enzyme in de novo purine biosynthesis and is involved in regulating chloroplast development and chlorophyll metabolism during leaf development (Zhang et al. 2018). Most of these mutants become green from the four-leaf stage. The *wsl9* mutant likewise gradually turned green from the four-leaf stage under field conditions. Transcription by NEP and PEP is a general mechanism of group-specific gene regulation during chloroplast development through recognition of distinct promoters (Hedtke et al. 1997). Previous studies showed that defects in PEP affect the development of chloroplasts and cause changes in leaf pigmentation. PEP-dependent genes (*psaA*, *psbA*, *rbcL*) were reduced in *wsl9* mutant at 20 °C which suggested that the *wsl9* mutant was defective in PEP activity under low temperature. This is consistent with phenotypes of the PEP-deficient mutants such as *wp1*, *wsl5,gars* (Wang et al. 2016; Liu et al. 2018; Cao et al. 2019). WSL9 is not the member of PEP complex and might not directly regulate the expression of photosynthesis-related genes. Down regulation of photosynthesis-related genes is caused by chloroplast development abnormality.

The editing efficiencies of *rpoB* at C467 and C560 were increased in *wsl9* mutant compared with WT. This phenotypic defect is most reminiscent of the previously reported *iojap* mutant in maize and *wsl4* mutant in rice in which the editing sites of *rpoB* are both highly edited (Halter et al. 2004; Wang et al. 2017). According to the previous reports, codon 127–299 and codon 779–802 of *rpoB* are located within Dispensable Region (Borukhov et al. 1991; Severinov et al. 1994), thus this Dispensable Region may be deleted without effect on the basic function of *E.coli* enzyme. It is possible that editing of the *rpoB* sites in Dispensable Region is non-essential (Corneille et al. 2000). C467 (codon 156) and C560 (codon 187) of *rpoB* are located within Dispensable Region. Thus the abnormal editing site of *rpoB* at Dispensable Region may not be responsible for the phenotype of *wsl9*.

*rpl2* encodes 50S ribosomal protein L2, and it is an crucial component of the translational apparatus in chloroplasts. Absence of this protein is a very sensitive marker for absence of ribosomal function, because it is involved in the peptidyl-transferase center (Nierhaus 1982). Therefore, low contents of L2 protein in *wsl9* under low temperature (Fig.8c) indicated that the defective *rpl2* splicing possibly caused ribosome-deficient plastids. Impairment of the translational apparatus then resulted in defective chloroplast development. The phenomenon of “*rpl2* not spliced” has also been found in the other white stripe mutants like *wsl, wsl4* (Tan et al. 2014; Wang et al. 2017). Under low temperature, the rRNAs, including 23S and 16S rRNAs, were decreased in *wsl9* (Fig. 6e, f). The absence of ribosomal protein RPL2 and rRNAs would obviously cause defects in ribosome biosynthesis, previous studies have shown that defects in the biogenesis of chloroplast ribosomes result in severe chlorotic phenotypes during early leaf development (Schmitz-Linneweber et al. 2006; Ostheimer et al. 2003; Song et al. 2014; Wang et al. 2016). We therefore propose that the mutation of *WSL9* results in defective ribosome biogenesis under low temperature, which ultimately chloroplast development during early seedling growth. However, it is possible that there is a unknown mechanism that *WSL9* regulates chloroplast development at low temperature, which will be our next research focus.

RNA-seq analysis was performed on *wsl9* and wild-type plants grown at 20 °C and 30 °C to study the molecular mechanism of *WSL9* in regulating chloroplast development under different temperature conditions. Our findings showed that under low temperature *WSL9* regulates expression of genes, involved in carbohydrate metabolic processes, thylakoid membrane organization, ATP binding, oxidation-reduction process, chloroplast development (Additional file 9: Figure. S6). Many genes involved in regulating carbon dioxide process were dramatically changed in *wsl9* at low temperature such as *OsLOX8*, *OsLOX9* indicating that mutation in *wsl9* leads to defects in photosynthesis in young plants. RNA-seq data showed that the expression of *WSL9* is decreased at 20 °C compared with 30 °C. Previous studies showed that not all Chl-deficient mutants are to be temperature-sensitive. *TCD9* encodes a Cpn60 protein (*tcd9*) mutant, which exhibited the albino phenotype under low temperature whereas displayed normal green under high temperature; but the expression of *TCD9* has no difference between 20 °C and 30 °C in wild type plants (Jiang et al. 2014). *YSS1* encodes a chloroplast nucleoid-associated protein required for chloroplast development in rice seedlings, the phenotype of *yss1* mutant is severe at low temperature, but the expression of *YSS1* is decreased at 20 °C compared with 30 °C (Zhou et al. 2016).

## Conclusions

The *WSL9* gene encodes a novel protein with an HNH motif. Disruption of *WSL9* led to a white-striped seedling phenotype in the field, and temperature-sensitive phenotypes under controlled conditions. Further studies are required to uncover the role of *WSL9* in chloroplast development.

## Methods

### Plant Materials and Growing Conditions

The *wsl9* mutant was isolated from an ethylmethane sulfonate (EMS) mutagenesis mutant pool of *japonica* cultivar Ninggeng 3. Seedlings for studies on temperature effects were grown in a growth chamber with a 16 h light/8 h photoperiod and constant temperatures of 20 °C, 25 °C, and 30 °C. Nearly all analyses used third leaves at about 10 days post-germination.

### Measurement of Chlorophyll Contents

Fresh leaves were collected and used to determine chlorophyll contents using a spectrophotometer and a previously described method (Arnon 1949). About 0.2 g of fresh leaves were collected in 10 ml tubes, 5 ml of 95% ethanol was added and tubes were held in darkness for 48 h. Supernatants were then collected following centrifugation and analyzed with a DU 800 UV/Vis 102 Spectrophotometer (Beckman Coulter) at 665, 649 and 470 nm, respectively.

### Transmission Electron Microscopy

Leaves from WT and *wsl9* seedlings for TEM analysis were cut into small pieces, fixed in 2.5% glutaraldehyde in phosphate buffer at 4 °C for 4 h, rinsed, and incubated overnight in 1% w/v OsO_4_ at 4 °C. The tissues were then dehydrated in an ethanol series and embedded in Spurr’s medium prior to thin sectioning. The samples were examined with a Hitachi H-7650 transmission electron microscope.

### Map-Based Cloning of the *WSL9* Allele and Complementation of *wsl9*

Genetic analysis was performed on F_2_ populations from reciprocal crosses Ninggeng 3 × *wsl9* and *wsl9* × Ninggeng 3. An F_2_ mapping population was constructed from the cross *wsl9* mutant × 93–11; 768 plants with the *wsl9* phenotype were used for fine mapping. New SSR/InDel markers were developed based on the Nipponbare and 93–11 (*indica*) genome sequences (http://www.gramene.org/). Primers were designed with Primer Premier 5.0. The PCR-based molecular markers used in the study are listed in Additional file 1: Table S1. The PCR cycling protocol comprised an initial denaturation step (95 °C/5 min), followed by 35 cycles of 94 °C/30 s, 55 °C/30 s, and 72 °C/34 s, with a final extension step of 72 °C/5 min.

### Complementation Tests

The *WSL9* locus was narrowed to a 89 kb genomic region flanked by InDel markers N12 and N3–11 on the long arm of chromosome 3 (Additional file 1: Table. S1). For complementation of the *wsl9* mutation, a ~ 2.1 kb upstream sequence and a 564 bp WT CDS fragment were amplified from the WT (primer CWSL9 pairs and PWSL9 (Additional file 3: Table S3)), and cloned into binary vector pCAMBIA1390 to generate a pCAMBIA1390-*WSL9* vector*.* This plasmid was introduced into *wsl9* mutants by agroinfection (Hiei et al. 1994).

### Sequence and Phylogenetic Analyses

Gene prediction and structure analysis were performed using the GRAMENE database (www.gramene.org/). Homologous sequences of *WSL9* were identified using the Blastp search program of the National Center for Biotechnology Information (NCBI, www.ncbi.nlm.nih.gov/). Multiple sequence alignments were conducted with DNAMAN. A phylogenetic tree was constructed using MEGA7 software.

### RT-PCR and Quantitative Real-Time PCR (qRT-PCR)

Total rice RNA was extracted with an RNA prep pure plant kit (TIANGEN, Beijing). The cDNA first strand was reverse-transcribed using oligo(dT) as primer. qRT–PCR was conducted using an ABI7500 real-time PCR system with the SYBR Green MIX in three biological repeats. Gene-specific primers used in real-time PCR are listed in Additional file 2: Table S2. The rice *Ubiquitin* gene was used as an internal control.

### RNA Analysis

Total RNA was isolated from third leaves of WT and *wsl9* seedlings. RNA samples were diluted to 10 ng/mL and analyzed using an Agilent 2100 analyzer. An RNA 6000 Nano Total RNA Analysis kit (Agilent) was used to measure concentrations.

### RNA Editing Sites

Specific cDNA fragments were generated by PCR amplification following established protocols with the respective primers (Takenaka and Brennicke 2007). The cDNA sequences were compared. The primers used for RNA editing analysis were obtained as reported previously (Tan et al. 2014).

### RNA Splicing Analysis

The chloroplast gene with at least one intron was selected and amplified using RT–PCR with primers flanking the introns. The primers used for RNA splicing analysis are listed in Table S2.

### RNA-Seq Analysis

RNA-sequencing (RNA-seq) analyses were performed on an Illumina Hiseq2000/2500 (LC Sciences) following the vendor’s recommended protocol and single end sequencing was performed on an Illumina Hiseq2500 instrument (OE Biotech, Shanghai). Significantly differentially expressed genes were identified based on a *P*-value of ≤0.05 and a log2 fold-change of (log2_FC) ≥2. Ontology analyses of these genes were carried out by referring to GOseq (Young et al. 2010). Pathway enrichment analyses were conducted using the Kyoto Encyclopedia of Genes and Genomes (KEGG) database (Kanehisa et al. 2008).

## Supplementary information

**Additional file 1: Table S1.** Newly designed PCR primers used for gene mapping.

**Additional file 2: Table S2.** Primers for quantitative real-time PCR, RNA splicing.

**Additional file 3: Table S3.** Primers used for vector construction.

**Additional file 4: Figure S1.** Phylogenic analysis of WSL9. a Structure of WSL9. b Evolutionary analysis of WSL9 and its homologs. c Alignment of amino acid sequences with highest identity to WSL9. Red arrow indicates the position of amino acid change in the wsl9 mutant.

**Additional file 5: Figure S2.** Expression profiles of the *WSL9* gene from http://ricexpro.dna.affrc.go.jp/.

**Additional file 6: Figure S3.** Editing efficiencies of *rpoB* genes in WT, *wsl9* mutant and complemented plants at different temperature. RT-PCR products of *rpoB* transcripts of WT, *wsl9* mutant, and complemented plants (*WSL-com*) grown in a growth chamber were sequenced. Editing efficiencies of *rpoB* at C467 and C560 in *wsl9* mutant were significantly increased compared to WT and complementation plants (Com) at 20 °C. Green, black, red, and blue peaks represent A, G, T, and C, respectively. Red boxes indicate editing sites.

**Additional file 7: Figure S4.** Expression levels of chlorophyll synthesis genes in wild type and *wsl9* mutant. (Student’s *t*-test, ∗∗, *P* < 0.01).

**Additional file 8: Figure S5.** RNA-seq analysis of WT and *wsl9* seedlings at 30 °C. mRNA was enriched from total RNA isolated from 10-d-old WT and *wsl9* mutant seedlings using oligo-(dT) fragmented and reverse-transcribed using random hexamer primers. The library was then constructed and sequenced using an Illumina HiSEquation 2000. **a** Frequencies of detected genes sorted according to expression level. **b** Read numbers of WT and *wsl9* mutant sequences. **c** Volcano plot showing overall alterations in gene expression in WT and *wsl9* mutant.

**Additional file 9: Figure S6.** RNA-seq analysis of WT and *wsl9* mutant grown at 20 °C and 30 °C. **a** Up-regulated differentially expressed genes comparing M2 and W2 and M3 and W3. **b** Down-regulated differentially expressed genes for M2-vs-W2 and M3-vs-W3. **c** Go analysis of genes differentially expressed between M2 and W2. **d** Go analysis of genes differentially expressed for M3-vs-W3. W3 and W2 represent WT plants grown at 30 °C and 20 °C, respectively. M3 and M2 represent *wsl9* mutant plants grown at 30 °C and 20 °C, respectively.

## Data Availability

All data supporting the conclusions of this article are provided within the article (and its additional files).

## Abbreviations

WT: Wild type
GO: Gene ontology
qRT-PCR: Quantitative real-time polymerase chain reaction
RNA-seq: RNA sequencing
TEM: Transmission electron microscopy
