# Supplementary material for: WSL9 Encodes an HNH Endonuclease Domain-Containing Protein that Is Essential for Early Chloroplast Development in Rice
Source: Rice (N Y). 2020 Jul 11;13:45. doi: 10.1186/s12284-020-00407-2 (PMC7354284; doi:10.1186/s12284-020-00407-2)
Supplement: Supplementary file 1 — Additional file 1: Table S1. Newly designed PCR primers used for gene mapping. [file 12284_2020_407_MOESM1_ESM.docx]

**Additional file 1:**

**Table S1.** Newly designed PCR primers used for gene mapping.

| Primer name | Forward primer (5'-3') | Reverse primer (5'-3') |
| --- | --- | --- |
| InDel 3-2 | TACTTTAATTTTGCAGCTC | TTTTACCCCACTCCATCT |
| InDel 3-6 | GTTTACGAATGAACCAGT | CTCATTGAGGCAAAGGAC |
| InDel 3-7 | CTGCACCGGAGAAATTTGAT | CGCATGCAGATGAATAGGTG |
| InDel 3-9 | CAGGCCGGATCTAGTTGAAA | CAAAGTGAACAGGCTCGAGAT |
| WF-9  N1 | CTGCAGTTACAAATCAAATCC  CGGTGCCTTTTTCTCCTCCT | TTAACTCGTAAAGTTGAACGT  GCACGGGTCTACAGCTAGTA |
| N4 | TTAATTTGTTTCCGGCGCCT | AGGATAATCGCCATGCTGGA |
| N11 | TCCTCCCTAAAACAGAGCCC | ACAACAAATGTGGCTGTCCA |
| N12 | GTGCATGTGCCTTGCTAGAA | AAAACCCCAAAATCAACTCCAAA |
| N3-11 | AAAGTGTTGGTGAGCATAGC | TTTGTGTTTGGAGAGACGAG |
| N3-12 | GTCCAATGATTCGTTCCCAC | CTTCACCGTTCACCAATTCC |
| dCAPs | CAGGGTGGGAAGAGCACATTGGAGGAAT | TTGTGACTGAAGAGAGGCGC |
