# Supplementary material for: WSL9 Encodes an HNH Endonuclease Domain-Containing Protein that Is Essential for Early Chloroplast Development in Rice
Source: Rice (N Y). 2020 Jul 11;13:45. doi: 10.1186/s12284-020-00407-2 (PMC7354284; doi:10.1186/s12284-020-00407-2)
Supplement: Supplementary file 2 — Additional file 2: Table S2. Primers for quantitative real-time PCR, RNA splicing. [file 12284_2020_407_MOESM2_ESM.docx]

**Additional file 2:**

**Table S2**. Primers for quantitative real-time PCR, RNA splicing

| Primer name | Forward primer (5'-3') | Reverse primer (5'-3') |
| --- | --- | --- |
| UBQ | ACCCTGGCTGACTACAACATC | AGTTGACAGCCCTAGGGTG |
| WSL6-RT | TTGTAGGGTCTCAGGACGTG | TTGGATTCTGCAACCACCTG |
| HEMA-RT  HEML-RT  HEMB-RT  HEMC-RT  URO-D-RT  HEMF-RT  CHLD-RT  CHLH-RT  CHLI-RT  CHLM-RT  CRD-RT  DVR-RT  POR-RT  CHLG-RT  *RPOB*  *atpF*  *ndhA*  *ndhB*  *petB*  *petD*  *rpl2*  *rpl16*  *rps16*  *trnA*  *trnG*  *trnI*  *trnK*  *trnL*  *trnV*  *ycf3-1*  *ycf3-2*  *rps12*  *23S* | GATGCAATCACTGCTGGAAAGCGT  AGAACAAAGGGCAGATTGCTGCTG  TGGCATTGTCAGGGAAGATGGAGT  TCATTCCGAGGGCTATTGGCTTCA  AGGCTTCCACTGACAGGTGTTGAT  ACTGACTGCACGATGGCAGTATGA  TAGCACAGCTGTCAGAGTGGGTTT  GCACGGGAACTTGGCGTTTCATTA  AGGGATGCTGAACTCAGGGTGAAA  GCTTCATCTCCACGCAGTTCTACT  TGGATCTAACATGACACGCACCCA  TTCTTCGAGAGGGTGATCAGGGAA  TCGTCGGCCTCGTCTGAGTTTATT  CCAGCCACTGATGAAAGCAGCAAT  GTCCTGGTATTTACTACCGC  ATGAAAAATGTAACCCATTCTT  ATGATAATAGACAGGGTACAGG  ATGATCTGGCATGTACAGAATG  TTCTCATATACGGTTCTCGG  ATGGGAGTAACAAAGAAACC  ACGGCGAAACATTTATACAA  ATGCTTAGTCCCAAAAGAAC  AAAACGATGTGGTAGAAAGC  GGGGATATAGCTCAGTTGGT  TCGTTAGCTTGGAAGGCTAG  TGGGCCATCCTGGACTTGA  GGTTGCCCGGGACTCGAA  GGATATGGCGAAATCGGTA  TAGGGCTATACGGATTCGAA  TGATAAGACCTTCTCAATTGTAGCC  AGAGCATACAAAGGCTTTGGAAT  ACTATCAACCCCAAAAAACC  TTCAAAAGAGGAAAGGCTTG | CCATCTTGCCAGCACCAATCAACA  TGTTTCGTCAAGTCACGGAGAGCA  CCAAAGCAGCACGTATTGCTCCAA  ACACTCTAGTTGGCCAATGGTGGA  AAAGAACGCCAGGGTCAACATTCC  AGAGATCGAGCCATTCCTTTGGGT  TTGCCAGCCACCTCAAGTATCTCA  ACATGTCCTGGAGCTGCTTCTCAT  AAGTAGGACTCACGGAACGCCTTT  GCAATGACGAATCGAAGACGCACA  ACTGTAACGGCATTCTTCTCCGGT  GAAACTGGCAATGGCAGCCAAGAA  AGGCCTCTCTCACTGAAAGCTGAA  AGAGCGCTAATACACTCGCGAACA  TCCCCACCTACACAAGCAAA  TTCATCGCCCTTTGTTTTTC  TTATAGTGAAACAAGTTGGGAAG  CTAAAAGAGGGTATCCTGAGCA  TAAAGGGCCCGAAATACCTT  TGTTGCTCCAATACCTAACC  TTACTTACGGCGACGAAGAATA  AACCGAAGAAATTGACTTCG  AGAATTCCGCCTTCCTTAAA  TGGAGATAAGCGGACTCGAA  GCGGGTATAGTTTAGTGGTA  AGCTCAGTGGTAGAGCGCG  GGGTTGCTAACTCAATGGTAGAG  TGGGGATAGAGGGACTTGA  AGGGCTATAGCTCAGTTCGG  GTGTGTATAAGGCCTATGTTATAGAGT  TTCAACCAGTTCTGTGCTTCAATATA  TTTGGCTTTTTGACCCCAT  AGAGAGCACTCATCTTGGGG |
