# Supplementary material for: WSL9 Encodes an HNH Endonuclease Domain-Containing Protein that Is Essential for Early Chloroplast Development in Rice
Source: Rice (N Y). 2020 Jul 11;13:45. doi: 10.1186/s12284-020-00407-2 (PMC7354284; doi:10.1186/s12284-020-00407-2)
Supplement: Supplementary file 3 — Additional file 3: Table S3. Primers used for vector construction. [file 12284_2020_407_MOESM3_ESM.docx]

Additional file 3:

Table S3. Primers used for vector construction.

| Purpose | Forward primer (5'-3') | Reverse primer (5'-3') |
| --- | --- | --- |
| Complementation construction | Pwsl9-F  Pwsl9-R  Cwsl9-F  Cwsl9-R | CCGGCGCGCCAAGCTTGTCAAGCCTAGCAAGATGTG  CCCACCATTATCTCCCTATCTCCTCG  GGGAGATAATGGTGGGGAGGAAGCCGCT  GAATTCCCGGGGATCC TCATTGGATTCTGCAACCAC |
