# Supplementary material for: WSL9 Encodes an HNH Endonuclease Domain-Containing Protein that Is Essential for Early Chloroplast Development in Rice
Source: Rice (N Y). 2020 Jul 11;13:45. doi: 10.1186/s12284-020-00407-2 (PMC7354284; doi:10.1186/s12284-020-00407-2)
Supplement: Supplementary file 4 — Additional file 4: Figure S1. Phylogenic analysis of WSL9. a Structure of WSL9. b Evolutionary analysis of WSL9 and its homologs. c Alignment of amino acid sequences with highest identity to WSL9. Red arrow indicates the position of amino acid change in the wsl9 mutant. [file 12284_2020_407_MOESM4_ESM.docx]

**Additional file 4:**

**Figure S1**


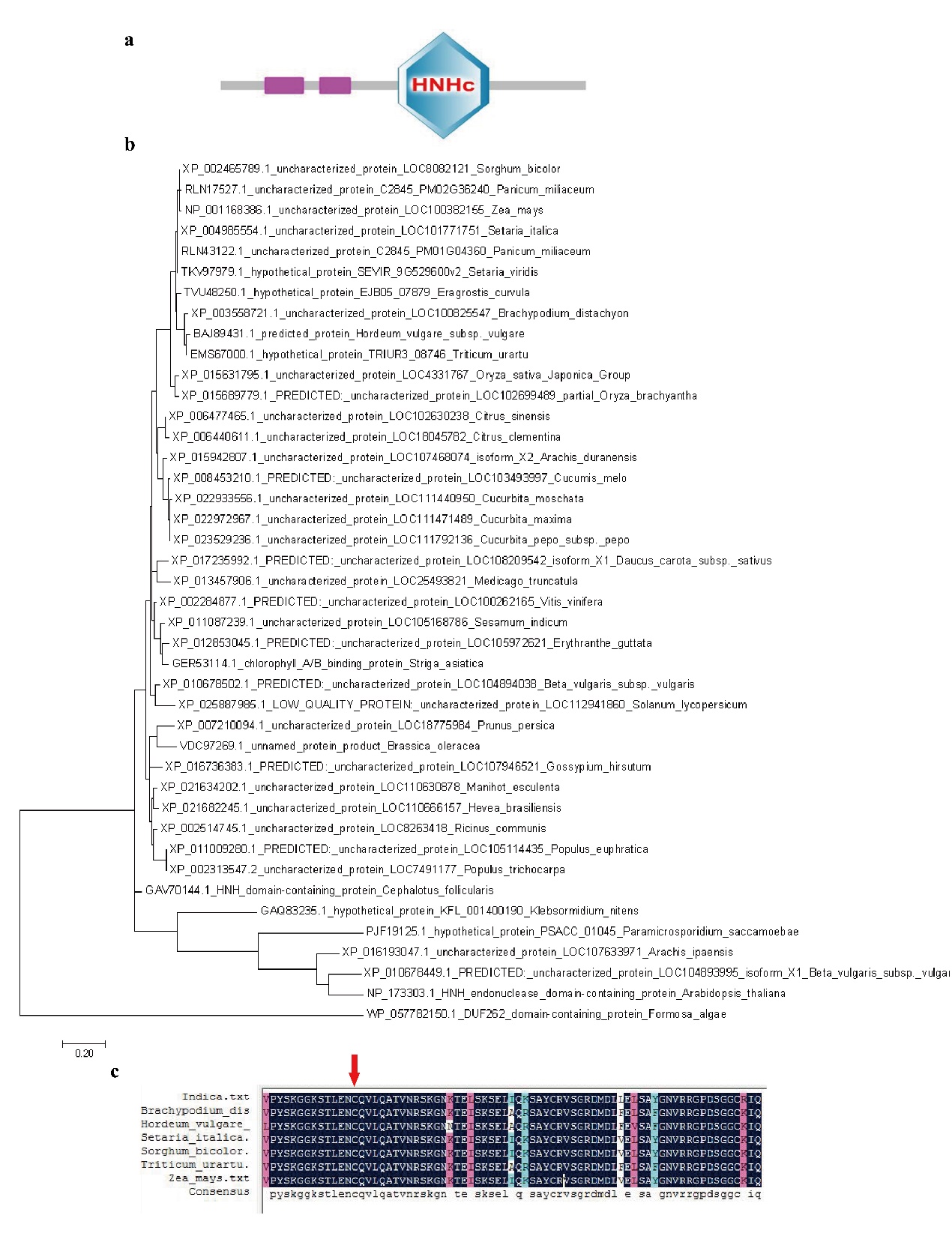


**Figure S1** Phylogenic analysis of WSL9. a Structure of WSL9. b Evolutionary analysis of WSL9 and its homologs. c Alignment of amino acid sequences with highest identity to WSL9. Red arrow indicates the position of amino acid change in the wsl9 mutant.
