# Supplementary material for: WSL9 Encodes an HNH Endonuclease Domain-Containing Protein that Is Essential for Early Chloroplast Development in Rice
Source: Rice (N Y). 2020 Jul 11;13:45. doi: 10.1186/s12284-020-00407-2 (PMC7354284; doi:10.1186/s12284-020-00407-2)
Supplement: Supplementary file 5 — Additional file 5: Figure S2. Expression profiles of the WSL9 gene from http://ricexpro.dna.affrc.go.jp/. [file 12284_2020_407_MOESM5_ESM.docx]

**Additional file 5:**

**Figure S2**


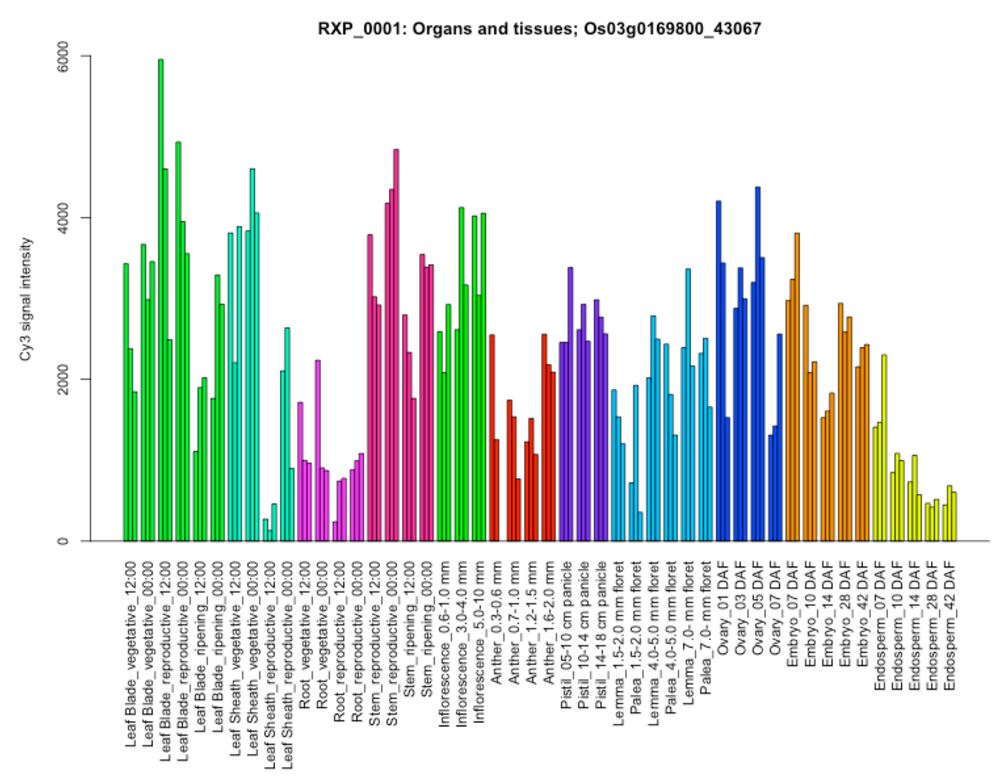


**Figure S2** Expression profiles of the *WSL9* gene from http://ricexpro.dna.affrc.go.jp/.
