# Supplementary material for: WSL9 Encodes an HNH Endonuclease Domain-Containing Protein that Is Essential for Early Chloroplast Development in Rice
Source: Rice (N Y). 2020 Jul 11;13:45. doi: 10.1186/s12284-020-00407-2 (PMC7354284; doi:10.1186/s12284-020-00407-2)
Supplement: Supplementary file 6 — Additional file 6: Figure S3. Editing efficiencies of rpoB genes in WT, wsl9 mutant and complemented plants at different temperature. RT-PCR products of rpoB transcripts of WT, wsl9 mutant, and complemented plants (WSL-com) grown in a growth chamber were sequenced. Editing efficiencies of rpoB at C467 and C560 in wsl9 mutant were significantly increased compared to WT and complementation plants (Com) at 20 °C. Green, black, red, and blue peaks represent A, G, T, and C, respectively. Red boxes indicate editing sites. [file 12284_2020_407_MOESM6_ESM.docx]

**Additional file 6:**

**Figure S3**


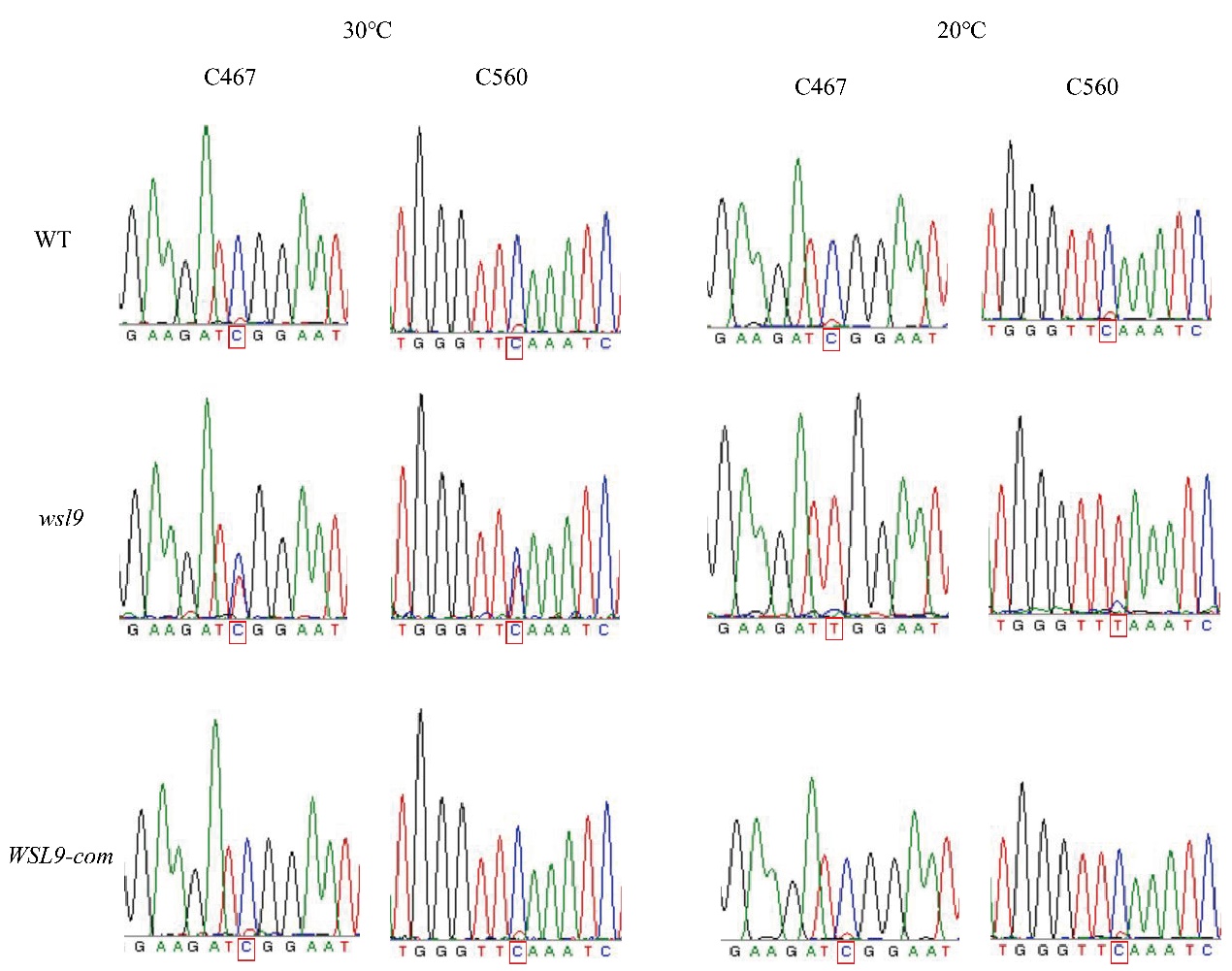


**Figure S3** Editing efficiencies of *rpoB* genes in WT, *wsl9* mutant and complemented plants at different temperature. RT-PCR products of *rpoB* transcripts of WT, *wsl9* mutant, and complemented plants (*WSL-com*) grown in a growth chamber were sequenced. Editing efficiencies of *rpoB* at C467 and C560 in *wsl9* mutant were significantly increased compared to WT and complementation plants (Com) at 20℃. Green, black, red, and blue peaks represent A, G, T, and C, respectively. Red boxes indicate editing sites.
