# Supplementary material for: WSL9 Encodes an HNH Endonuclease Domain-Containing Protein that Is Essential for Early Chloroplast Development in Rice
Source: Rice (N Y). 2020 Jul 11;13:45. doi: 10.1186/s12284-020-00407-2 (PMC7354284; doi:10.1186/s12284-020-00407-2)
Supplement: Supplementary file 7 — Additional file 7: Figure S4. Expression levels of chlorophyll synthesis genes in wild type and wsl9 mutant. (Student’s t-test, ∗∗, P < 0.01). [file 12284_2020_407_MOESM7_ESM.docx]

**Additional file 7:**

**Figure S4**


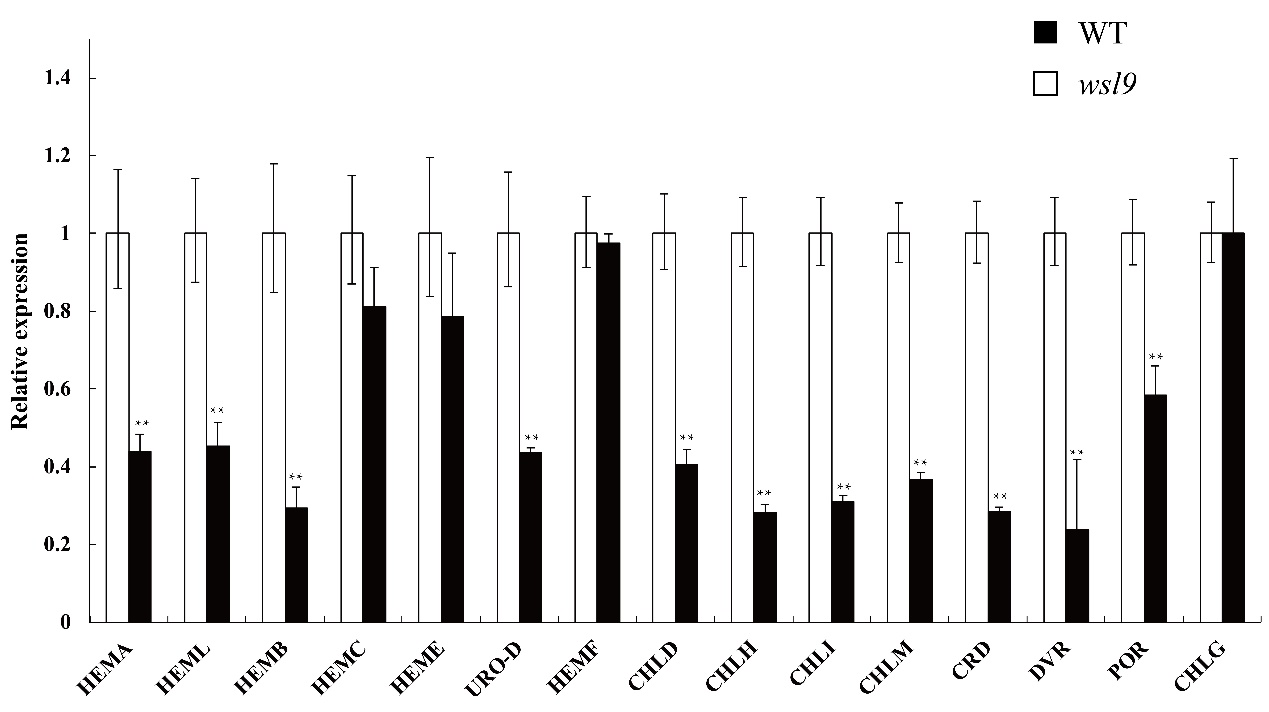


**Figure S4** Expression levels of chlorophyll synthesis genes in wild type and *wsl9* mutant (Student’s *t*-tests, ∗∗, *P* <0.01).
