# Supplementary material for: WSL9 Encodes an HNH Endonuclease Domain-Containing Protein that Is Essential for Early Chloroplast Development in Rice
Source: Rice (N Y). 2020 Jul 11;13:45. doi: 10.1186/s12284-020-00407-2 (PMC7354284; doi:10.1186/s12284-020-00407-2)
Supplement: Supplementary file 8 — Additional file 8: Figure S5. RNA-seq analysis of WT and wsl9 seedlings at 30 °C. mRNA was enriched from total RNA isolated from 10-d-old WT and wsl9 mutant seedlings using oligo-(dT) fragmented and reverse-transcribed using random hexamer primers. The library was then constructed and sequenced using an Illumina HiSEquation 2000. a Frequencies of detected genes sorted according to expression level. b Read numbers of WT and wsl9 mutant sequences. c Volcano plot showing overall alterations in gene expression in WT and wsl9 mutant. [file 12284_2020_407_MOESM8_ESM.docx]

**Additional file 8:**

**Figure S5**

**
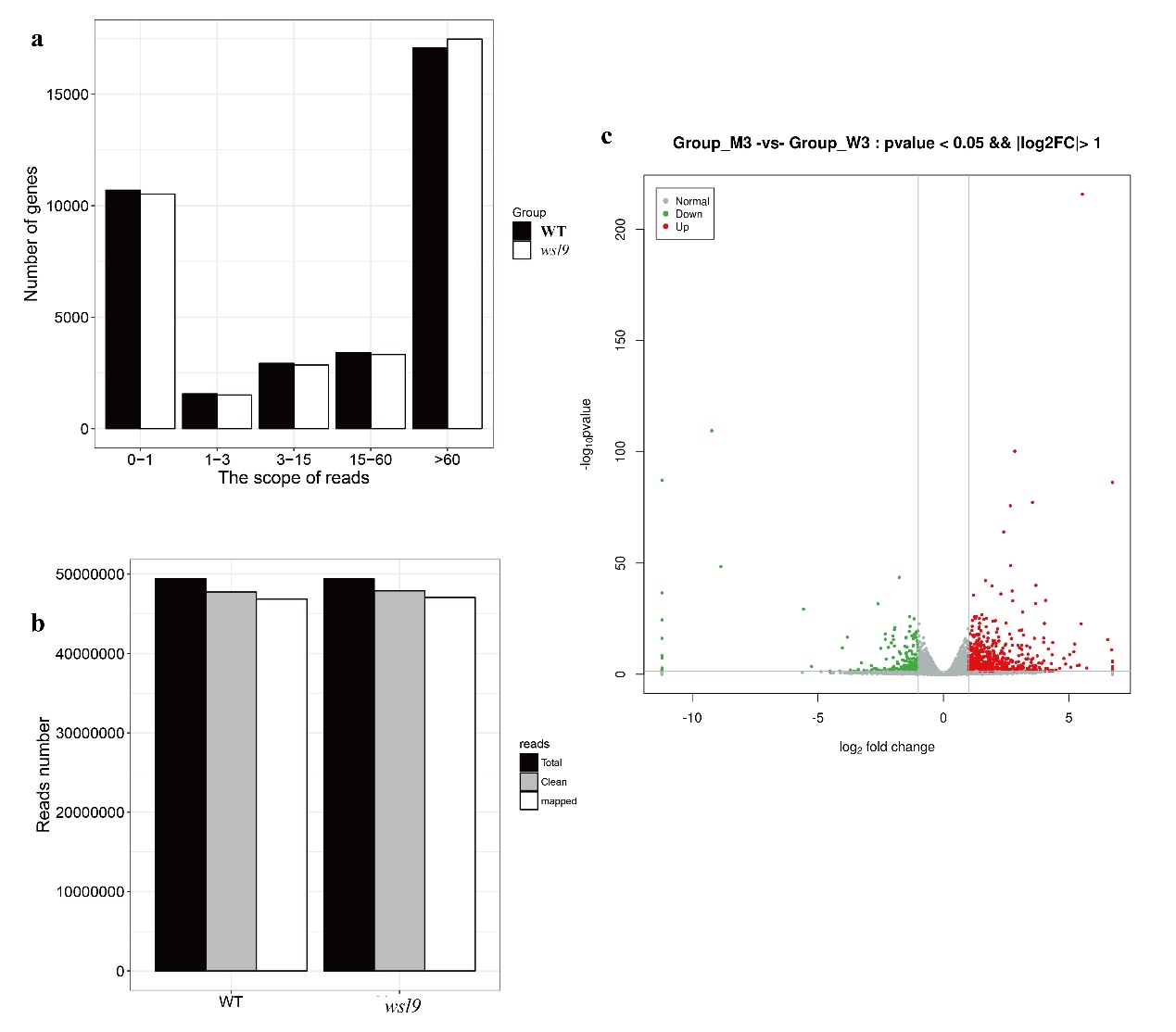
**

**Figure S5** RNA-seq analysis of WT and *wsl9* seedlings at 30℃. mRNA was enriched from total RNA isolated from 10-d-old WT and *wsl9* mutant seedlings using oligo-(dT) fragmented and reverse-transcribed using random hexamer primers. The library was then constructed and sequenced using an Illumina HiSEquation 2000. a Frequencies of detected genes sorted according to expression level. b Read numbers of WT and *wsl9* mutant sequences. c Volcano plot showing the overall alterations in gene expression in WT and *wsl9* mutant b- Y-AXIS ‘Read number’*.*
