# Supplementary material for: WSL9 Encodes an HNH Endonuclease Domain-Containing Protein that Is Essential for Early Chloroplast Development in Rice
Source: Rice (N Y). 2020 Jul 11;13:45. doi: 10.1186/s12284-020-00407-2 (PMC7354284; doi:10.1186/s12284-020-00407-2)
Supplement: Supplementary file 9 — Additional file 9: Figure S6. RNA-seq analysis of WT and wsl9 mutant grown at 20 °C and 30 °C. a Up-regulated differentially expressed genes comparing M2 and W2 and M3 and W3. b Down-regulated differentially expressed genes for M2-vs-W2 and M3-vs-W3. c Go analysis of genes differentially expressed between M2 and W2. d Go analysis of genes differentially expressed for M3-vs-W3. W3 and W2 represent WT plants grown at 30 °C and 20 °C, respectively. M3 and M2 represent wsl9 mutant plants grown at 30 °C and 20 °C, respectively. [file 12284_2020_407_MOESM9_ESM.docx]

**Additional file 9:**

**Figure S6**


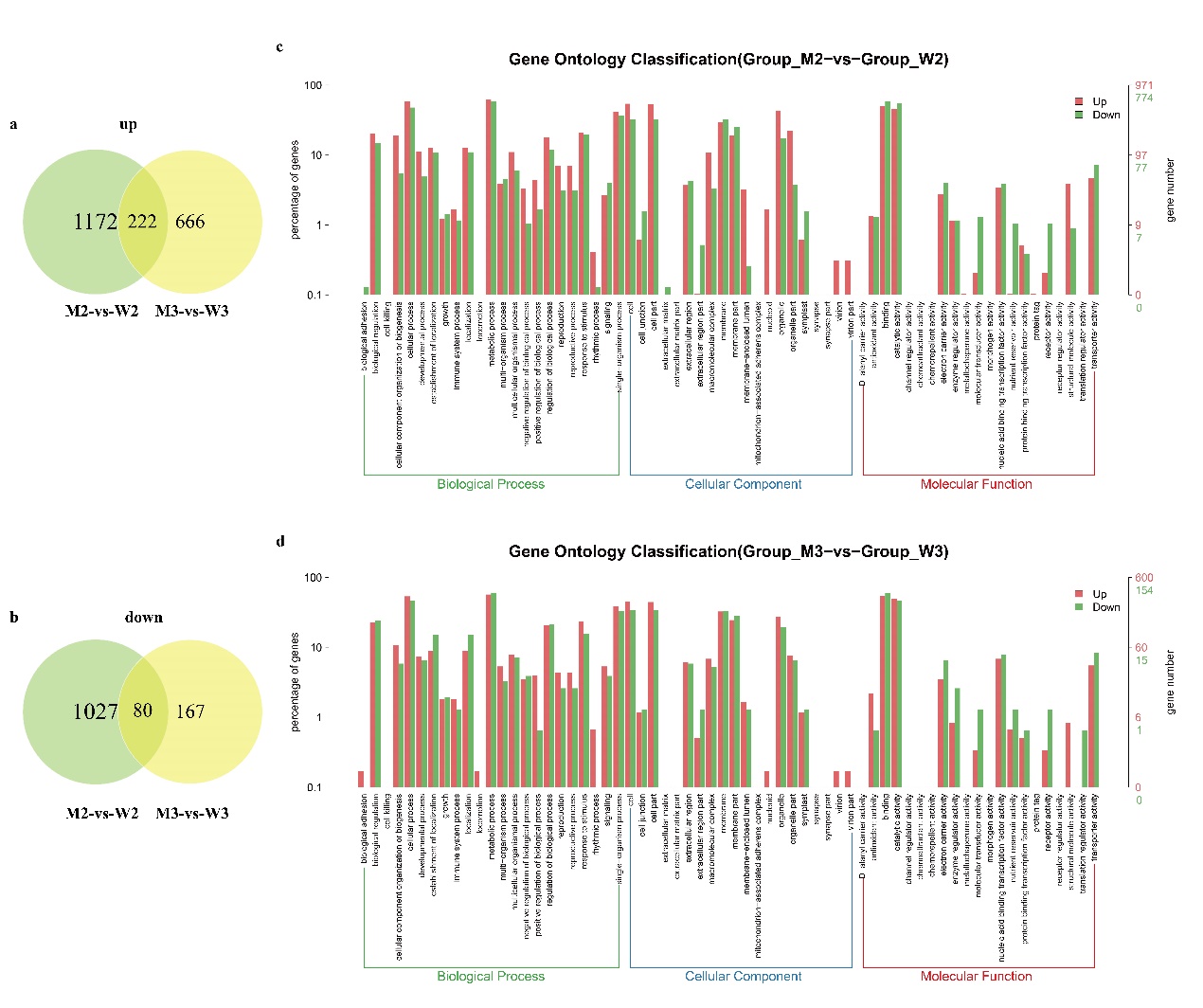


**Figure S6** RNA-seq analysis of WT and *wsl9* mutant grown at 20℃ and 30℃. **a** Up-regulated differentially expressed genes comparing M2 and W2 and M3 and W3. **b** Down-regulated differentially expressed genes for M2-vs-W2 and M3-vs-W3. **c** Go analysis of genes differentially expressed between M2 and W2. **d** Go analysis of genes differentially expressed for M3-vs-W3. W3 and W2 represent WT plants grown at 30℃ and 20℃, respectively. M3 and M2 represent *wsl9* mutant plants grown at 30℃ and 20℃, respectively.
